# Dietary patterns among adults in three low-income urban communities in Accra, Ghana

**DOI:** 10.1371/journal.pone.0293726

**Published:** 2023-11-09

**Authors:** Sandra Boatemaa Kushitor, Deda Ogum Alangea, Richmond Aryeetey, Ama de-Graft Aikins

**Affiliations:** 1 Department of Community Health, Ensign Global College, Kpong, Ghana; 2 Department of Food Science and Centre for Complex Systems in Transition, Stellenbosch University, Stellenbosch, South Africa; 3 Department of Population, Family and Reproductive Health, School of Public Health, University of Ghana, Legon, Accra, Ghana; 4 Institute of Advanced Studies, University College London, London, United Kingdom; Wroclaw University of Environmental and Life Sciences: Uniwersytet Przyrodniczy we Wroclawiu, POLAND

## Abstract

**Objective:**

Dietary patterns describe the dietary behaviour and habits of individuals. Unhealthy dietary patterns provide individuals with limited nutrients while increasing the risk of nutrition-related diseases. Unhealthy dietary patterns are high in urban areas, especially among low-income urban residents. This study examined dietary patterns in three low-income urban communities in Accra, Ghana, between 2011 and 2013.

**Methods:**

This study used Wave 2 and 3 data from the Urban Health and Poverty Survey (EDULINK 2011 and 2013). The sample size was 960 in 2011 and 782 in 2013. Dietary pattern was examined using factor analysis and the NOVA food classification system. Summary statistics were computed for sociodemographic characteristics and diet frequency and pattern. Differences in dietary behaviours between 2011 and 2013 were also estimated. Three logistic regression models were computed to determine the predictors of dietary patterns.

**Results:**

The frequency of consumption of animal-source foods (ASF) and fruits was higher in 2013 compared with 2011. The intake of processed culinary ingredients (NOVA Group 2), processed foods (NOVA Group 3) and ultra-processed foods (NOVA Group 4) was higher in 2013 versus 2011. In 2013, 29% consumed ultra-processed foods compared to 21% in 2011. Three dietary patterns (rice-based, snack-based, and staple and stew/soup) were identified. About two out of every five participants consumed the food items in the rice (43%) and staple and sauce patterns (40%). The proportion of participants who consumed the food items in the snack pattern was 35% in 2011 but 41% in 2013. Respondents aged 25–34 and those with higher education often consumed the snack-based and rice-based dietary patterns. In 2013, participants in Ussher Town had a higher probability of consuming food items in the snack pattern than those living in Agbogbloshie.

**Conclusions:**

This study found that between 2011 and 2013, more participants consumed ASFs, fruits, and processed foods. A complex interplay of personal and socio-cultural factors influenced dietary intake. The findings of this study mirror global changes in diet and food systems, with important implications for the primary and secondary prevention of NCDs. Health promotion programs at the community level are needed to address the increasing levels of processed food consumption.

## 1. Introduction

Dietary patterns describe the quantity, diversity, and frequency of different foods and beverages consumed in a population and the habits associated with these dietary characteristics [1]. Dietary patterns show all foods and beverages a person habitually consumes, which affects the person’s health [2]. These patterns are driven by individual choices influenced by multiple drivers, including dietary knowledge, social networks, and environmental factors such as food supply, food processing/preparation technologies, and food affordability [3].

In order to classify dietary habits as healthy or unhealthy, dietary patterns have been measured using dietary indices and data-driven analysis [4]. Diet indices such as the Healthy Eating Index (developed by the United States Department of Agriculture), the Healthy Diet Indicator (developed by the World Health Organisation), and the NOVA classification system have been designed as a guide in preventing and managing NCDs. The data-driven methods include factor analysis, cluster analysis and reduced rank regression [5, 6]. Both methods have produced dietary patterns that can be described as healthy or unhealthy depending on the combination of food items consumed. Healthy dietary patterns are meals that provide adequate macronutrients and micronutrient needs [2]. Such patterns usually include fruits, vegetables, nuts, whole grains and legumes and are associated with a reduced risk of diseases. Unhealthy dietary patterns include meal combinations high in salts, sugars, oils and refined grains [7].

Globally, dietary patterns are changing rapidly; the observed changes mirror the so-called nutrition transition characterised by increasing consumption of diets with limited dietary quality [7, 8]. Consumption of sugar-sweetened beverages, salt, red meat, and processed meat exceeds the recommended daily allowance [9]. These changes have been associated with individual and environmental factors stipulated by the socio-ecological framework [10, 11].

Individual-level factors related to dietary behaviours include socio-demographic characteristics (e.g., age, sex, and educational status), self-efficacy and cognition. (e.g. attitudes, knowledge, taste and preferences [12]. Environmental-level factors include social, physical, and macro-level influences. The social system of an individual, such as family, peer groups, and friends, provides social support and norms that influence dietary behaviours. The physical environment examines the availability and accessibility of food products within a given setting. For example, unhealthy dietary patterns are higher in urban areas compared with rural areas. The urban food environment is increasingly characterised by easier access to energy-dense street foods, processed foods, and fast foods. In low-income urban areas, access to calorie-rich processed foods is increasing due to the relatively lower prices and the convenience of such foods [13]. The macro-level describes the influence of public policies from government systems, organisations, businesses, and industries defining access to healthy foods and societal beliefs and diet perceptions. Therefore, the socio-ecological model was applied in this study to understand the individual and environmental factors that influence an individual’s food choice.

In Ghana, dietary pattern analysis indicates a high prevalence of unhealthy eating habits and low dietary diversity [14–16]. Among school children, an unhealthy dietary pattern characterised by consuming processed meat, fried foods and soft drinks was associated with increased obesity [17]. Ghanaian adolescents consume one or more portions of sugar-sweetened beverages daily–a higher level of consumption than the recommended dietary allowance [18]. In 2014 and 2015, about 53% of Ghanaian adults did not eat adequate servings of fruits and vegetables [19]. Furthermore, experts have noticed increased availability and consumption of polished rice, maize, frozen chicken, vegetable oil, and sugar nationally [20], especially in Accra [21].

Even though unhealthy food consumption practices are problematic in urban Ghana, large-scale population-based diet research has focused on rural populations and mostly on children. Also, dietary studies which generate longitudinal data on adults are almost non-existent in Ghana [22]. Longitudinal data involves data collection for two or more time points. Longitudinal study designs are essential to assessing dietary change and disentangling complex diet/disease relationships, essential for understanding population health patterns [23]. For example, in a systematic review of research conducted between 1998 and 2017, the authors noted that the increase in obesity in Ghana and Kenya could be explained by the low consumption of fruits and vegetables but the widespread intake of sugar-sweetened beverages [22]. Unfortunately, little is known about changes in dietary patterns using longitudinal data among adults in urban areas in Ghana. Therefore, the researchers recommended longitudinal studies in Africa on dietary behaviours among adult urban residents [22].

This study was designed to answer the question of dietary patterns and quality changes among adults in urban poor communities. The findings will inform the implementation and evaluation of evidence-based dietary interventions, including the recent dietary guidelines for Ghanaians published by the Ministry of Food and Agriculture [24]. The findings are also of practical use in health care settings, especially by providing information on patterns of diet that persist over time and can be targeted for interventions.

This study was conducted in Ga Mashie (James Town and Ussher Town) and Agbogbloshie, a low-income community in Accra, Ghana. This low-income urban community has an obesogenic food environment [25–27]. An earlier study reported discordance between their dietary knowledge and practice [25]. Furthermore, the communities have previously been identified as obesogenic, with limited fruit and vegetable vendors and high availability of convenience stores, street food, and alcohol vendors. Chronic non-communicable diseases (NCDs) are prevalent in the study area [27, 28]. Among the youth, the prevalence of pre-hypertension and hypertension was 32% and 4%, respectively, in 2011 [29]. Yet, people living with NCDs in the community cannot manage their conditions adequately and, thus, often experience complications [28]. The incidence of NCDs has been partly attributed to poor dietary practices and the lack of understanding of dietary behaviours linked with NCDs [25, 30].

In order to supplement existing information and thus fill the gap, this current study was directed to examine diet frequency and quality in Ga Mashie (James Town and Ussher Town) and Agbogbloshie in Accra, Ghana, using a repeated cross-sectional survey. The specific objectives were to 1) determined changes in selected dietary behaviours between 2011 and 2013; 2) determined changes in diet quality between 2011 and 2013 using NOVA, and 3) examined the factors associated with dietary pattern.

## 2. Materials and methods

### 2.1 Source of data

The current analyses used data from the second and third wave surveys of the European Union Cooperation Programme in Higher Education (EDULINK) Urban Health and Poverty Project, led by the Regional Institute for Population Studies of the University of Ghana [29, 31]. This data comes from a longitudinal study of health, poverty, and climate change further details about the EDLINK project can be found in Afrifa-Anane and colleagues [29]. Data on diet frequency were not collected in Wave 1. The second wave was conducted in 2011 (November to December), and the third wave in 2013 (September to October). The datasets will be referred to as 2011 for Wave 2 and 2013 for Wave 3.

The EDULINK project was a collaboration between the Ga Mashie Development Agency (GAMADA) and the local chief’s council. As form of reciprocity in each locality, gifts (household plastic wares) were given to the households that had been interviewed. This was done after data collection was completed for the entire community. The director of GAMADA, a youth activist and three chiefs reviewed the questionnaire, facilitated community meetings and were involved in the design of interventions that resulted from the EDULINK project. The EDULINK field team of 58 consisted of 23 RIPS affiliates (students, research assistants and national service personnel) and 37 hired professional fieldworkers (ten were local residents recommended by GAMADA). The fieldworkers were grouped into four teams of fifteen members. At least eight persons in the survey team spoke the local dialect Ga–necessary for speaking to residents in Ga Mashie. During training for the survey, the team broke up into local Ghanaian language groups after the questionnaires had been discussed in English, discussed translations of questions and informed consent into the various local languages, and agreed on standard ways of translating questions and presenting concepts in the local languages.

In 2011, residential structures were selected proportional to size using a two-stage sampling approach. The Ghana Statistical Service has previously demarcated the area into 80 enumeration areas (E.A.s): 48 in Usher Town, 24 in James Town, and 8 in Agbogbloshie [27]. Twenty-eight of these E.A.s were systematically sampled: Agbogbloshie (n = 5), James Town (n = 8) and Ussher Town (n = 16). All the housing structures in the selected E.A.s were listed to create a database of residents in the community. Forty residential structures were systematically determined from each E.A. A total of 974 individuals were interviewed from the 40 residential structures. In 2013, the 40 structures chosen in 2011 were re-sampled. Due to the high migration rates in the communities and attrition, some of the households and individuals in the 2011 selected structures were unavailable to participate. The total number of individuals who responded to the 2013 survey was 782. The response rate was 64% in 2011 and 62% in 2013. In 2013, about 80% of those who refused to participate were unavailable during the survey period, 15% had relocated, and 5% had died.

The data in 2011 and 2013 were used to create a repeated cross-sectional dataset. Only about 20% of the 2011 respondents were interviewed in 2013 (n = 194). Among the 20% who could form a panel dataset, about 8% had incomplete data (n = 16). The research team was left with a sample size of 178. The data analysis showed that 60% of the 178 were individuals aged 15–24 and did not reflect a representative sample of the community. Therefore, the research team decided to analyse the data as a repeated cross-sectional study. Repeated cross-sectional datasets allow for the analysis of change over time at the population level but not at the individual level [32]. The research team present the analysis of dietary frequency and pattern by year. However, the dietary pattern analysis was conducted on the pooled dataset.

### 2.2 Study variables

#### The NYU-UG Twin Cities food frequency questionnaire

Diet frequency was assessed using a food frequency questionnaire (FFQ) developed for a previous project, the New York University-University of Ghana (NYU-UG) project titled “*Assessing the Dietary Patterns and Health Status of Ghanaians in Accra and New York City”* [33]. This FFQ was modified by expert review and pretesting to include foods consumed by residents in the study. The final FFQ included 87 foods classified into 14 groups: cereal-based porridges; cereal-based staples; tuber and plantain-based staples; soups; stews/soups/ fats and oils; animal source foods; baked/roasted/boiled snacks; fried snacks; soft drinks; alcoholic drinks; milk and dairy products; fruits; and vegetables (S1 Table in S1 File). Specific food items were included in each food group; for example, the food items for the milk and dairy group were milk (evaporated or fresh), yoghurt/ice cream, butter, cheese/wagashie and others. The FFQ was used to record the frequency and source of consumption of the listed foods for the seven days preceding the survey; portion size consumed was not estimated. In addition to the listed foods, an opportunity was given to respondents to indicate foods they had consumed but were not listed in the FFQ. The respondents’ other food items were all recoded as part of the listed categories since they were like the existing list. For example, Fante Kenkey was recoded as Banku/Akple/T.Z./Kenkey. The FFQ was used to measure dietary frequency and dietary patterns. Two dietary behaviour variables were assessed: cooking frequency at home and salt use at the table. The frequency of cooking at home was reported as “never, daily, 1–2 days per week or 3–6 days per week”. Also, the frequency of adding salt to food at the table was assessed on a three-point scale: never, occasionally/rarely, or often.

#### Socio-demographic characteristics

Since dietary behaviours are grounded in socio-ecological conditions and realities, this study selected age, sex, and level of education as risk factors at the individual level. Ethnicity and marital status were selected as interpersonal risk factors. The locality of residence was selected as a community-level risk factor. These variables were chosen to examine how they are linked with dietary intake frequency, pattern, and quality. The age of participants was measured in completed years and categorised into 10-year age groups: 15–24, 25–34, 35–44 and 45+ years. Sex was categorised as 1 for females and 0 for males. Ethnicity was measured as Ga, Akan, Ewe, Mole-Dagbani, and non-Ghanaians. Participants who self-reported as Nigerian, Zabarama, Hausa and Fulani were classified as ‘non-Ghanaians.’ However, the non-Ghanaians have lived in the communities for decades and have contributed to the multi-cultural/multi-ethnicity context of the area.

### 2.3 Study analytical approaches

Two approaches were used to examine the dietary pattern in this study: hypothesis-oriented analysis and data-driven analyses.

#### Hypothesis-Oriented analysis

This study used the NOVA classification system. The NOVA classification system was developed for the prevention and management of NCDs [15]. The NOVA system provides a rigorous format for classifying foods based on the level of processing and their impact on diet and health [15]. By classifying food items into four groups, the NOVA system can isolate ultra-processed foods that are nutritionally unbalanced, such as snacks, drinks and convenience foods formulated from food constituents [15]. The NOVA classification system is recognised and used by international organisations such as the World Health Organisation, Pan American Health Organisation and the Food and Agriculture Organisation to measure diet quality with NCDs [16, 17]. Researchers in different countries and socio-economic contexts have used it to understand dietary behaviours. These studies indicate that individuals with a higher intake of ultra-processed foods have low diet quality but an increased risk of overweight and obesity, non-communicable diseases, and mortality [18, 19].

Unprocessed and minimally processed foods such as fruits, vegetables, and whole-grain foods were classified as group one (S2 Table in S1 File). Group two food items were processed culinary ingredients, group three was accessed food, and group four were ultra-processed, industrially formulated foods and beverages. Details of these food items can be found in S2 Table in S1 File. The frequency of consumption of foods belonging to each of the four groups was categorised into less than once a week (≤1x/wk), two to three times a week (2-3x/wk), four to five times a week (4-5x/wk) and more than five times a week (>5x/wk).

#### Data-driven analysis

Factor analysis was used to identify the underlying dietary patterns for the pooled dataset. The principal component analysis included 62 food items listed in S3 Table in S1 File. The factors were rotated using the varimax rotation to obtain orthogonal (uncorrelated) factors. Six factors had eigenvalues >1. However, in the Scree plot, the first three components stood out before the point where the curve flattened when the elbow rule was applied. These three factors were also interpretable (S1 Fig in S1 File). The Kaisera-Mayera-Olkina ratio for the data used for this study was 0.830. Kaisera-Mayera-Olkina values between 0.8 and 1 indicate adequate sampling [34].

Three meaningful patterns were identified. The three factors explained 80% of the total variance in the dataset. Tabachnick and Fidell suggest that using an alpha level of .01 (two-tailed), a rotated factor loading for a sample size of at least 300 would need to be at least .32 to be considered statistically meaningful as a rule of thumb (2007). Although a factor loading of 0.5 is recommended, we used 0.32 because of interpretability. For example, In S3 Table in S1 File, cake (FL: 0.51), malt (FL: 0.40) and (FL: 0.48) had factor loadings close to 5. The other items had a value of 0.30. In Ghana, fizzy drinks are usually consumed with chips, cake, and meat pie. Therefore, food items with factors loading higher than 0.3 were used to label the dietary patterns to reflect the dietary behaviours in Ghana.

Individual factor scores for each isolated pattern were saved and used as participants’ scores. The mean score was used as the cut-off point. Participants whose scores were at or above the mean were categorised as 1, and below the mean were categorised as 0. Therefore, each participant had a score for the three dietary patterns.

### 2.4 Statistical analysis

The socio-demographic characteristics of the respondents were assessed using summary statistics, including frequencies and percentages. The frequency of consumption of the 14 food groups, cooking at home and salt use at the table for each survey year was estimated using crosstabulations with a Chi-square test for each survey year. The frequency of consumption of the NOVA food groups was assessed using crosstabulations with a Chi-square test for 2011 and 2013.

This study estimated each dietary pattern by the background characteristics of the respondents using crosstabulations with the Chi-square test. Three logistic regression models were conducted to determine the relationship between each dietary pattern and the background characteristics of respondents (age, sex, locality, level of education, study year and marital status). An interaction term for study year and locality of residence was added to each model to account for economic conditions prevailing within each community during the survey years. About 5% of the variability in the data was explained by staple and rice pattern models. The snack pattern model had an. All the models were specified correctly based on the linear predicted value (_hat) and the linear predicted value square (_hat). For example, the snack pattern had a linear predicted value coefficient of 1.03 with a p-value of 0.000 and the linear predicted value squared was 0.024 with a p-vale of 0.871. The analyses were conducted using Stata 17. The p-values reported were two-tailed, and a cut-off of <0.05 was considered for statistically significant associations.

## 3. Results

### 3.1 Socio-demographic characteristics 2011 and 2013

The current analysis includes data from 960 participants from 2011 and 782 from 2013 who had complete data on all the relevant variables of interest (Table 1). In both surveys, about one-third of participants were aged between 15 and 24 years. Almost half had Middle/JHS level education (44% and 42%, from 2011 and 2013, respectively). About half lived in Ussher Town, and a third in James Town. Two out of five participants were married in both survey waves. The majority were Ga-Dangme.

**Table 1:** Demographic characteristics of respondents, 2011 and 2013.

Variable	2011 (n = 960)			2013 (n = 782)		
	Female	Male	Total	Female	Male	Total
**Age**						
15–24	32.5	34.5	33.4	35.8	38.0	36.6
25–34	32.5	30.5	31.6	33.9	26.6	30.7
35–44	23.8	17.8	21.0	22.4	16.1	19.5
45+	11.2	17.1	14.0	7.8	19.3	13.2
**Level of education**						
No education	8.3	3.8	6.2	9.7	3.8	7.0
Primary	26.2	12.9	20.2	20.7	11.4	16.6
Middle/JHS	44.6	43.2	44.0	41.6	43.7	42.5
Secondary	18.5	32.4	24.8	23.9	34.1	28.5
Higher	2.4	7.7	4.8	4.1	7.0	5.4
**Locality of residence**						
Agbogbloshie	18.0	16.0	17.1	13.7	13.7	13.7
James Town	30.5	33.3	32.0	26.0	30.2	27.9
Ussher Town	51.6	50.7	50.9	60.3	56.1	58.4
**Marital Status**						
Never married	33.8	17.9	39.6	39.9	44.8	42.1
Currently married	46.9	47.0	44.4	45.0	42.2	43.8
Currently not married	19.2	11.9	16.0	15.0	13.0	14.2
**Ethnicity**						
Akan	30.5	26.5	28.8	25.6	23.2	24.5
Ga-Dangme	53.7	61.7	57.2	56.9	65.2	60.5
Ewe	5.9	4.0	5.1	9.1	4.6	7.2
Mole-Dagbani	3.1	3.5	3.2	3.2	3.2	3.2
Non-Ghanaians	6.8	4.2	5.6	5.2	3.8	4.6

### 3.2 Diet frequency and behaviours

**Fig 1:**
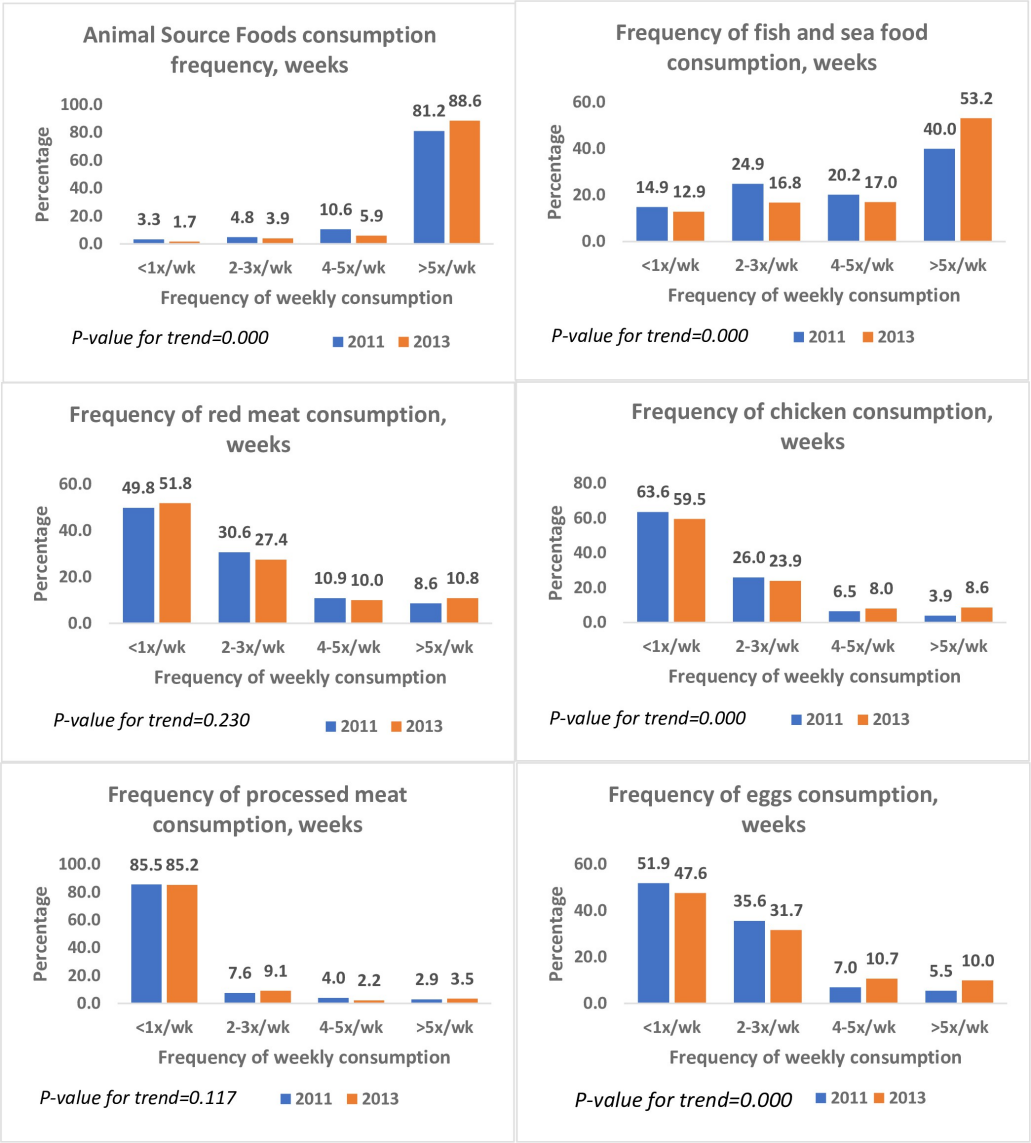
Frequency of consumption of animal source foods in Agbogbloshie and Ga Mashie, 2011 and 2013.

**Fig 2:**
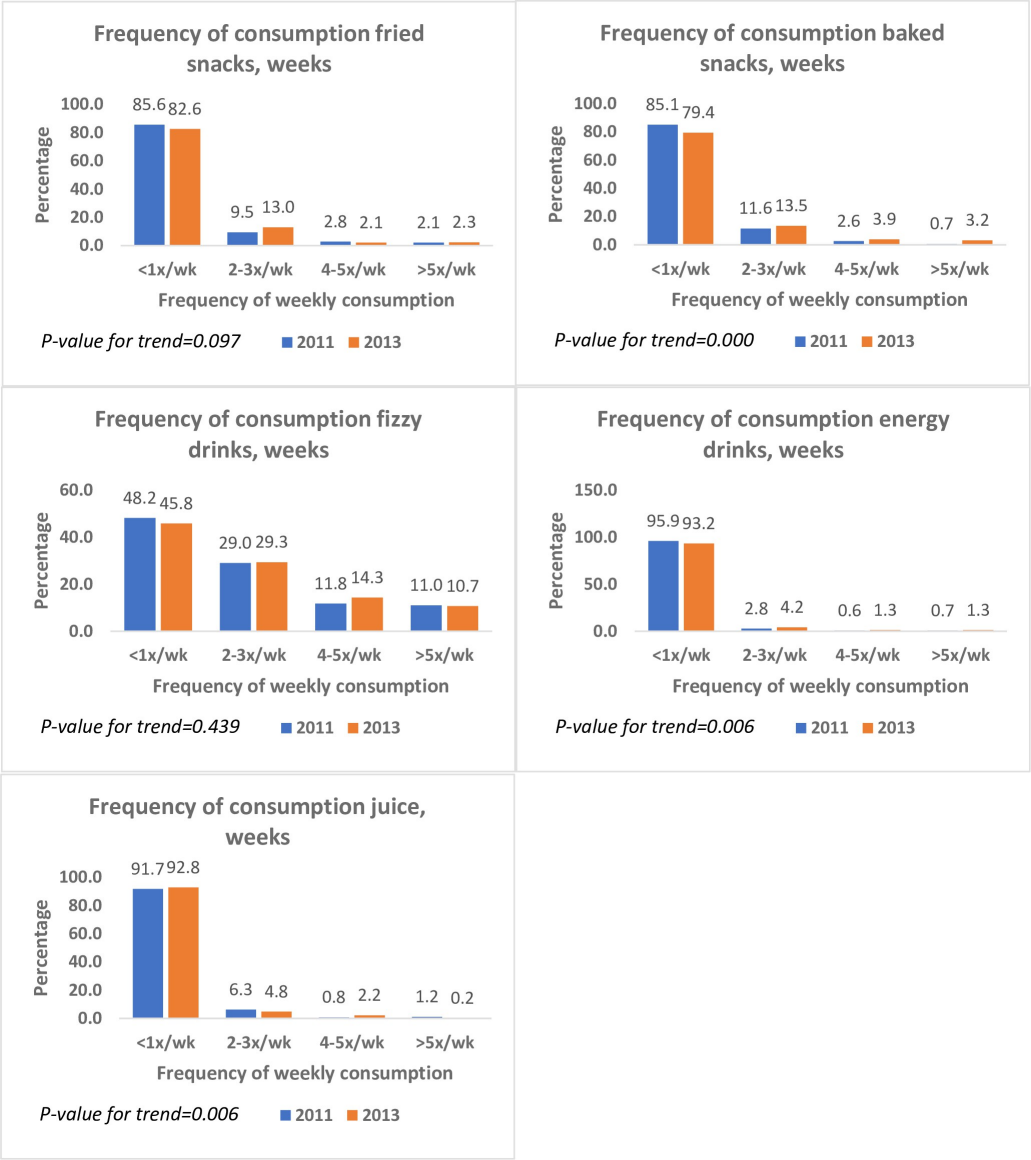
Frequency of snack foods consumed in Agbogbloshie and Ga Mashie, 2011 and 2013.

**Fig 3:**
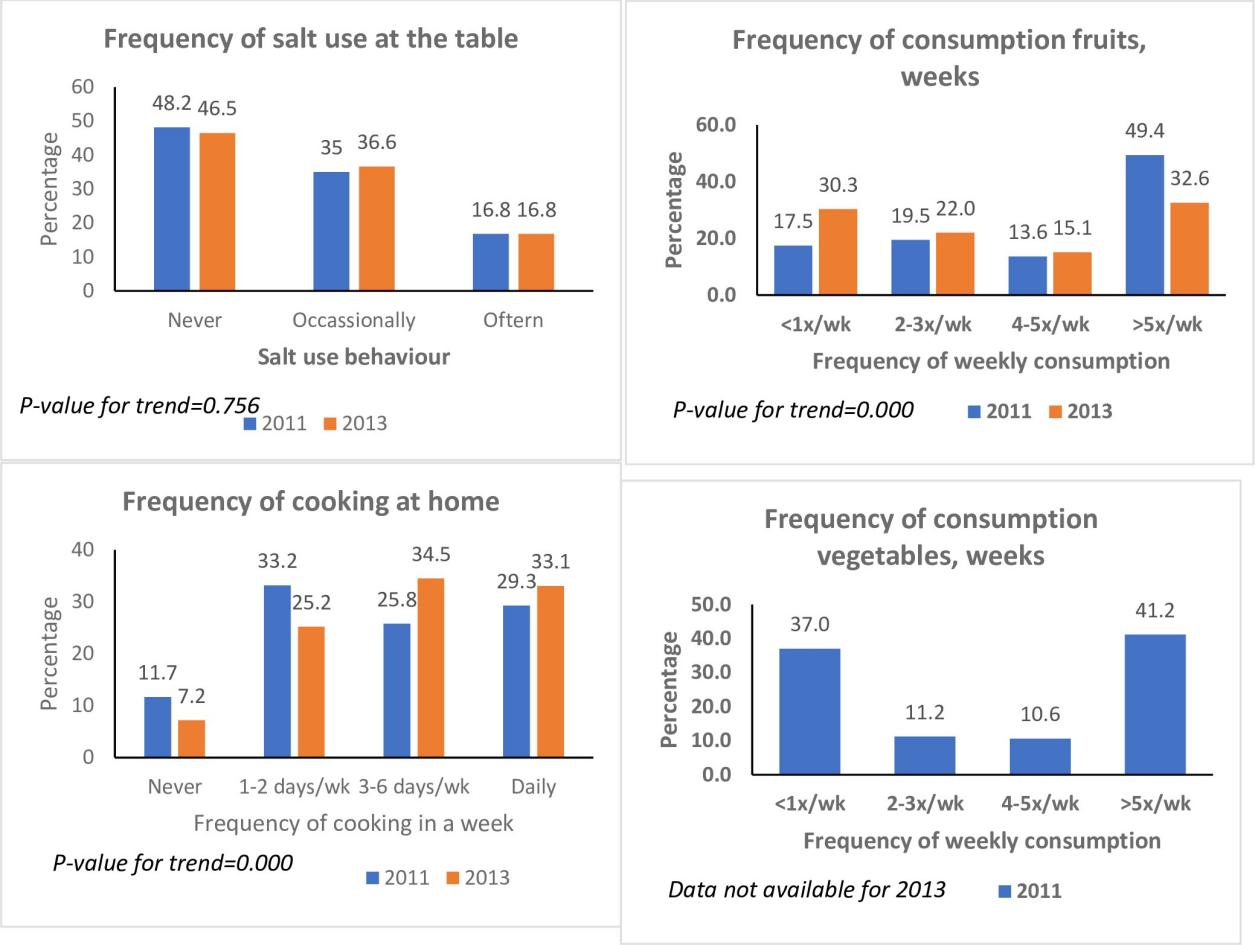
Selected dietary behaviours in Agbogbloshie and Ga Mashie, 2011 and 2013.

The frequency of consumption of animal-source foods in 2013 was higher than in 2011 (*P* = 0.000). The proportion of participants who consumed ASFs more than five times a week increased from 81% to 87% (Fig 1). Participants who consumed baked snacks five times or more weekly increased from 1% in 2011 to 3% in 2013 (Fig 2). Fizzy drinks were the single most frequently consumed snacks. About 11% consumed fizzy drinks five times or more per week in both surveys. In 2011, nearly two out of five respondents consumed vegetables five times or more weekly. The proportion of the sample who consumed fruits five times or more a week was 20% lower in 2013 compared with 2011. The frequency of using salt at the table was similar between 2011 and 2013 (Fig 3). Across both surveys, about a third of the participants cooked food at home daily (29% and 33%, respectively). The proportion who never cooked at home reduced from 12% in 2011 to 7% in 2013 (*P* = 0.000).

### 3.3 Diet quality using NOVA

Table 2 shows the respondents’ diet quality for 2011 and 2013, assessed using the NOVA classification framework. In 2011 and 2013, almost all the participants consumed Group 1 food items five times or more weekly. The Group 1 items included unprocessed or minimally processed foods such as kenkey, rice, and roasted maize. Gradual increase in consumption was observed for processed culinary ingredients (Group 2), processed foods (Group 3) and ultra-processed foods (Group 4) food items between surveys. The proportion that consumed Group 2 food items five times or more per week increased from 67% to 83%, and Group 4 food items from 21% to 29% between 2011 and 2013 (Table 2). Between 2011 and 2013, there was a 10% difference in the proportion of participants who consumed Group 3 food items five times or more in a week.

**Table 2:** Diet quality of participants by NOVA classification, 2011 and 2013.

NOVA groups	2011	2013	p-value for trend
**Consumed food from Group 1/week**			
<1 time	0.0	0.1	p<0.0001
2–3 times	0.1	0.1	
4–5 times	0.2	0.3	
>5 times	99.7	99.5	
**Consumed food from Group 2/week**			
<1 time	9.4	4.3	p<0.0001
2–3 times	12.2	6.0	
4–5 times	11.1	7.2	
>5 times	67.3	82.5	
**Consumed food from Group 3/week**			
<1 time	24.6	19.8	p<0.0001
2–3 times	28.0	20.4	
4–5 times	17.5	18.0	
>5 times	29.9	41.8	
**Consumed food from Group 4/week**			
<1 time	39.3	32.3	p<0.0001
2–3 times	26.5	23.7	
4–5 times	12.9	15.1	
>5 times	21.3	28.9	

### 3.4 Dietary pattern

The factor loadings of foods for the identified dietary pattern are presented in S2 Table in S1 File. Three dietary patterns were identified: rice, snack, and staple and stew/soup. Meals frequently consumed by the rice pattern were boiled rice, jollof rice, tomato stew, kenkey and shito (pepper sauce). The snack pattern was characterised by more frequent consumption of fruit juices, yoghurt, malt drinks, meat pie, cake, sausage, flour chips, doughnuts, meat pie, malt drinks, sugar-sweetened beverages, frozen dairy and bananas. The staple and stew/soup pattern was characterised by fufu, ampesi (boiled yam/plantain), palm soup, light soup, grounded pepper, kontomire (cocoyam leaves), palava sauce, and garden egg stew.

**Fig 4:**
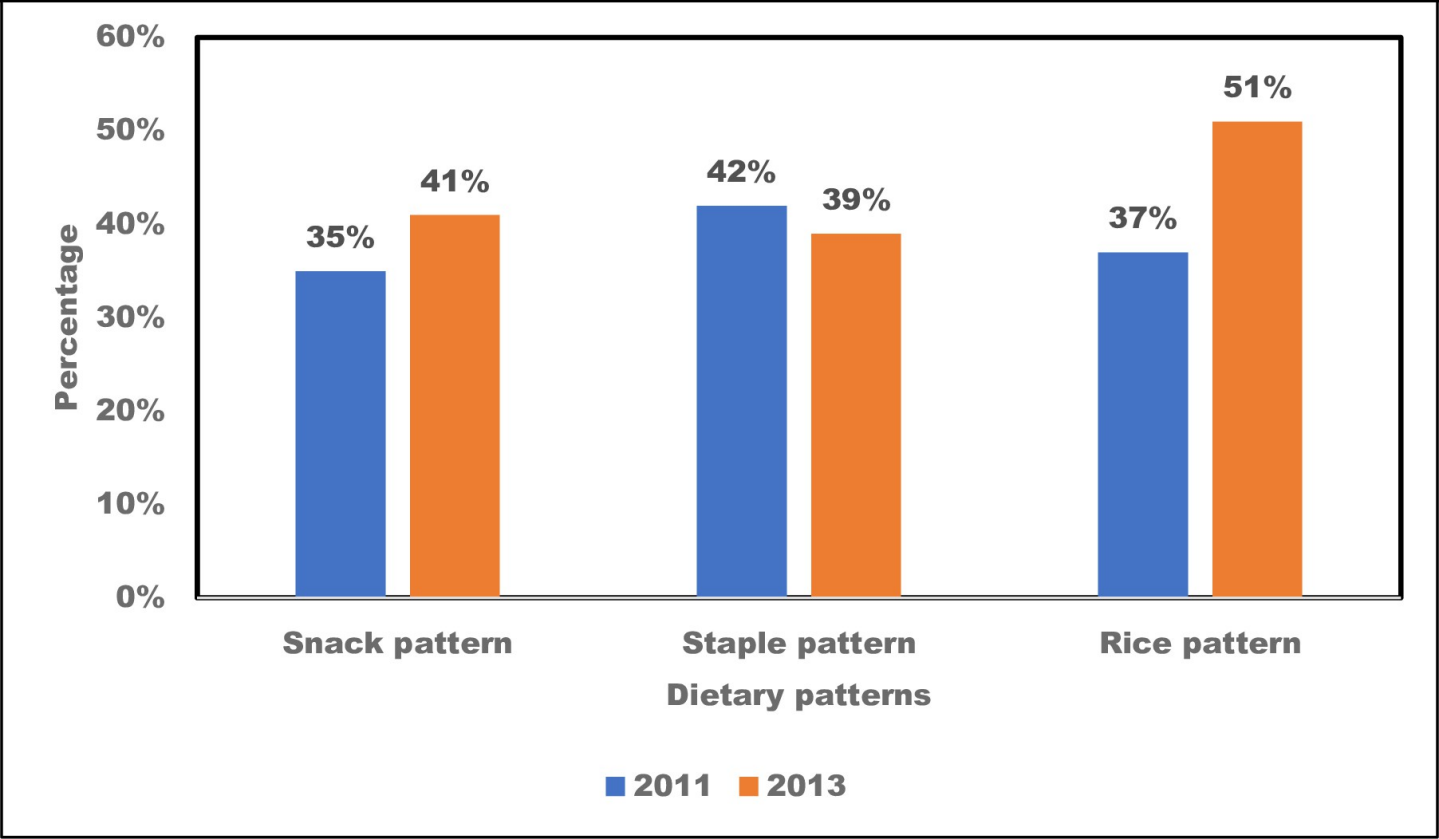
Dietary patterns in Agbogbloshie and Ga Mashie, 2011 and 2013.

In 2011, about two out of every five participants consumed food items in the rice (37%) and staple and sauce patterns (42%) (Fig 4). The proportion of participants who consumed the food items in the snack pattern was 35% in 2011 but ten points higher in 2013 (41%). The proportion of respondents who belonged to the rice dietary pattern increased between 2011 and 2013 (Fig 4). However, there was a reduction in the portion of those who consumed the staple pattern between 2011 and 2013.

There were significant associations between snack pattern and age (*p<*0.000), level of education (p = 0.001), and locality of residence (p<0.001) (Table 3). The snack pattern was about 70% less likely among those aged 35–44 years compared with those aged 15–24 years (p = 0.001) (Table 4). The odds of consuming the snack pattern were higher for respondents living in Ussher Town in 2013 than those living in Agbogbloshie. Locality of residence and sex were significantly associated with the rice pattern. Across localities, the rice pattern consumption was higher among participants in Ussher Town (OR = 2.61) and James Town (OR = 2.69) compared with Agbogbloshie. There was strong evidence of an association between age and the staple pattern. A higher proportion of those aged 25–34 years (34%), 35–44 years (51%), and 45+ years consumed food items in the staple pattern compared to those aged 15–24 years (29%). The pattern of individuals consuming food items in the staple pattern as age increases was maintained when other factors when controlled for Table 4.

**Table 3:** The relationship between characteristics of respondents and dietary patterns.

Characteristics of respondents	Snack dietary pattern			Rice dietary pattern			Staple dietary pattern		
	OR	95% CI	P	OR	95% CI	P	OR	95% CI	P
**Age**									
15–24									
25–34	0.971	0.72–1.32	0.848	0.968	0.72–1.29	0.830	1.793	1.31–2.44	0.000
35–44	0.690	0.51–0.94	0.018	0.766	0.56–1.04	0.092	2.401	1.74–3.29	0.000
45+	0.659	0.48–0.89	0.008	0.585	0.42–0.80	0.001	2.673	1.94–3.68	0.000
**Sex**									
Male									
Female	0.973	0.79–1.19	0.786	1.241	1.01–1.52	0.040	0.094	0.75–1.12	0.409
**Level of education**									
No education									
Primary	0.771	0.49–1.19	0.245	1.001	0.61–1.63	0.997	1.033	0.65–1.62	0.887
Middle/JHS	1.143	0.75–1.71	0.522	1.202	0.76–1.89	0.428	1.457	0.96–2.20	0.077
Secondary	1.268	0.82–1.95	0.285	1.282	0.79–2.06	0.304	1.093	0.70–1.70	0.693
Higher	2.634	1.37–5.03	0.003	1.298	0.70–2.40	0.406	0.823	0.45–1.50	0.526
**Locality name**									
Agbogbloshie									
James Town	1.069	0.71–1.59	0.743	2.696	1.62–4.46	0.000	0.473	0.31–0.70	0.000
Ussher Town	1.006	0.69–1.45	0.973	2.610	1.61–4.22	0.000	0.414	0.28–0.59	0.000
**Marital status**									
Never married									
Married	0.950	0.75–1.19	0.659	1.195	0.94–1.51	0.139	1.033	0.82–1.29	0.782
Currently not married	0.866	0.63–1.17	0.358	0.869	0.62–1.20	0.399	0.906	0.67–1.23	0.536
Year of study									
2011									
2013	0.643	0.387	0.089	2.165	1.17–3.99	0.014	1.029	0.61–1.71	0.913
Locality name#Year of study									
James Town#2013	1.151	0.620	0.656	0.825	0.40–1.67	0.595	1.708	0.91–3.17	0.091
Ussher Town#2013	1.784	1.008	0.047	0.753	0.38–1.46	0.403	1.100	0.61–1.95	0.746

**Table 4:** The distribution of dietary patterns by background characteristics of respondents.

Characteristics of respondents	Snack dietary pattern		Rice dietary pattern		Staple dietary pattern	
	n (%)	P	n (%)	P	n (%)	P
**Age**						
15–24	273 (45%)	0.000	284 (47%)	0.021	179 (29%)	0
25–34	212 (39%)		235 (43%)		229 (42%)	
35–44	100 (28%)		152 (43%)		181 (51%)	
45+	68 (29%)		81 (35%)		114 (49%)	
**Sex**						
Male	140 (41%)	0.869	189 (55%)	0.051	127 (37%)	0.33
Female	175 (40%)		209 (48%)		176 (40%)	
**Level of education**						
No education	35 (30%)	0.001	39 (34%)	0.012	50 (44%)	0.002
Primary	100 (31%)		126 (39%)		121 (37%)	
Middle/JHS	280 (37%)		323 (43%)		344 (45%)	
Secondary	202 (44%)		220 (48%)		160 (35%)	
Higher	38 (43%)		45 (51%)		30 (35%)	
**Locality name**						
Agbogbloshie	74 (27%)	0.000	80 (29%)	0.000	151 (21%)	0.000
James Town	202 (39%)		253 (48%)		220 (31%)	
Ussher Town	379 (40%)		420 (45%)		334 (47%)	
**Marital status**						
Never married	150 (45%)	0.062	182 (55%)	0.048	102 (31%)	0.000
Married	127 (38%)		171 (51%)		151 (45%)	
Currently not married	38 (35%)		45 (41%)		50 (46%)	

## 4. Discussion

This study used different indices to examine the dietary behaviour of residents in three urban communities in Accra, Ghana, using two surveys conducted in 2011 and 2013. Overall, dietary behaviour changed between 2011 and 2013. Intake of ASFs and fizzy drinks was higher in 2011 compared with 2013 (refer to Fig 2). When examined with factor analysis, these diet behaviours showed a pattern of higher rice and snack consumption but lower consumption of staple foods (refer to Fig 4). Regarding diet quality, there was a significant increase in the consumption of processed foods (refer to Table 2). In addition, trends in consumption varied by sex, age, locality, year of study, and level of education (refer to Table 4). The consumption of processed foods was more pronounced among the youth and persons with a higher level of education.

The results from other studies among urban residents across the globe showed a similar trend. In the United Kingdom, ultra-processed foods constitute about two-thirds of total energy intake [35]. In the USA [36] and Nigeria [37], urban residents consumed higher processed foods than rural residents. A 2013 survey in Kumasi, Ghana’s second-largest city, reported that about a quarter of respondents consumed ‘risky’ foods, similar to our findings (29%) (Refer to Table 2) [38]. Also, another study in Sekondi and Kumasi reported a decrease in the consumption of traditional snacks, drinks and main meals [39]. In the subsequent paragraphs, we explain our research findings using factors at the individual, community and structural levels outlined by the socio-ecological model [10].

At the individual level, the decrease in consumption of staple and stew/sauce pattern can be explained by concerns consumers have about the safety (physical and microbial), convenience (cooking time, packaging) and cost of these food items [39]. An analysis of the microbial quality of foods sold in formal and informal food outlets affirms this concern. In particular, soups, stew, fufu (cassava and plantain pudding), macaroni, vegetable salad and waakye (boiled rice with beans) were the most contaminated food samples [40]. Due to job demands, some urban residents have limited time to purchase and prepare food. In addition, the relatively high cost of some traditional foods also hinders their consumption [25]. The reverse is valid for the consumption of processed foods in various countries. Processed foods are relatively safe, convenient, and affordable.

We found that the young and those with higher education had a higher intake of processed foods than adults and those with less education. In Ghana, the level of education is rising, mainly among the youth. According to the 2014 Ghana Demographic and Health Survey, about one-tenth of young women and men (15–19 years) had no education, compared to nearly two-fifths of older women and men (45–49 years) [41]. This suggests that the highly educated youth are the population group adopting a lifestyle of consuming snacks and sweetened drinks. Therefore, this dietary behaviour could have been acquired during the schooling years of the respondents. In low-income urban communities, schools do not typically have canteens. Students frequently depend on street food vendors and convenience stores for food; these vendors typically sell snacks and sweetened drinks [42]. In the school environment, social desirability to adopt snacks and sweetened drinks among peers is expected, as reported in Ghana [43] and South Africa [44]. Furthermore, although this population are likely to have high nutrition knowledge, their knowledge may not translate into practice due to the discomfort and inconvenience of preparing and adopting healthy behaviours [14, 45].

Dietary habits are influenced by social, cultural and accessibility within a particular location [16]. While Agbogbloshie has the largest fresh produce market in Accra, increasing access to fruits and vegetables for its residents, Ga Mashie has community markets with limited vendors selling fruits and vegetables [27]. In Ga Mashie and Agbogbloshie, more than half of the population engages in sales/services such as petty trading, food vending, shopkeeping and bartending. They operate as mobile or stationary individual enterprises and provide services at least six days a week. These convenience stores stock limited healthy foods but high-sugar and fatty foods. The stores co-exist with alcohol stores and drinking bars, all within walking distance [28]. In a community with limited spaces and time to cook, the food vendors offer residents convenient access to unhealthy foods, and these outlets’ patronage is pretty high [46].

Two factors at the structural level can explain the study findings. Firstly, agricultural and trade policies implemented by the government have significantly changed the local food environment. Prominent among these policies include the structural adjustment policies and the promotion of a private sector-led economy in the 1980s [47]. These policies have facilitated the importation and domestic production of industrial snacks and beverages. In 2014, sugar was the fourth largest commodity imported into Ghana [48]. Both multinational and domestic companies have invested significant amounts of money into producing, distributing, and marketing carbonated soft drinks, energy, sports, and alcoholic drinks. In 2015, for example, about $100m was invested by a multinational company to improve the infrastructure and operations of a local brewery [49]. These investments have increased the availability of sugar-sweetened beverages at very competitive prices.

Secondly, there is a sub-optimal implementation of local and international policies to improve the food environment [50]. For example, Codex guidelines for nutrition labelling, false claims and hygiene are barely enforced [51]. Photographic adverts of sugar-sweetened beverages on significant highways in Accra contain health and fitness claims [52]. The WHO guidelines for marketing unhealthy foods to young children are also not enforced. In addition, information on the nutrition composition of food items does not exist even at multinational food outlets such as Kentucky Fried Chicken (KFC), Burger King, Pizza Inn, Chicken Inn, Pizza Hut, and Barcelos [50]. Yet, these outlets provide meals with a high energy content of between 1500 and 2000 kcal per serving [53]. Furthermore, recommendations to tax unhealthy food items such as alcohol, tobacco and soft drinks are yet to be implemented despite intense advocacy by civil society [54].

The findings demonstrate that the youth were consuming processed foods more frequently. Exposure to unhealthy foods during childhood and adolescence increases the risk of addiction to these foods. The increasing consumption of snacks and sweetened drinks among youth in Ghana will likely exacerbate the pattern of increased NCDs and associated complications and premature deaths. Furthermore, research on Ghanaian food habits suggests that people do not consider snacks as meals [55]. Therefore, they will be less likely to reduce their energy intake from main meals when consuming energy-dense snacks. This will result in the consumption of excess calories, leading to significant increases in weight gain over time, even among the physically active.

This study has some limitations. Firstly, the portion size of the foods consumed was not assessed. Secondly, the EDULINK surveys used a 7-day FFQ, a handy tool for determining usual dietary consumption; however, the tool may not accurately reflect long-term dietary behaviours. Despite these limitations, the study findings are not unique to Ghana. The results are a replica of global changes in diet and food systems, with important implications for the primary prevention of NCDs.

Based on a longitudinal study of NCD risk in the community, recommendations for improving NCD interventions, including dietary behaviours, were suggested [28]. For example, at the community level, it was recommended that public health education through local radio and television programs could be designed to provide residents with knowledge. Such programs can focus on the dangers of processed foods while promoting cooking and eating unprocessed foods.

There is an attempt at the structural/policy level to address aspects of dietary habits. The government has implemented regulations banning the importation and sale of turkey tails and marketing breast milk substitutes [50]. For instance, the ban on turkey tails has significantly reduced the availability of high-fat meats. Turkey tails, locally known as ‘*chofi*’ served with fried yam and or/and bread, were commonly consumed as street food. Food vendors publicly displayed and sold it along crucial stops on the Accra-Kumasi highway. In 1999, the government implemented a food policy that limited the amount of fat in beef, mutton, pork and poultry imported into the country. Through the joint efforts of the Food and Drugs Board (FDB), the National Security and the Customs, Exercise and Preventive Service (CEPS), the turkey tails smuggled into the country were seized and destroyed at the ports of entry before reaching the market [56]. The success of the turkey tail policy could be an opportunity to implement other regulations on the food environment, such as the 2019 National Policy for the Prevention and Control of NCDs.

## Conclusion

In a poor urban community in Ghana, this examined dietary behaviours between 2011 and 2013. The findings indicate a significant increase in the consumption of ASFs, fruits, and processed foods. This dietary behaviour was common among the youth and those with higher education. Poultry was the dominant ASF consumed. The findings of this study mirror global changes in diet and food systems, with important implications for the primary and secondary prevention of NCDs. Public health programs can focus on the regulation of the food environment in addition to promoting healthy eating behaviours. These health promotion programs can be implemented through community radios and durbars by the Ga Mashie Development Agency (GAMADA) and the Ussher Polyclinic Public Health Unit. This study was unable to gather information on portion size of the meals consumed. This limited the assessment of changes in macro and micronutrient intakes over time. Future studies can use local modules to gather data on portion sizes to estimate macronutrients and micronutrients intake.

## Supporting information

S1 File (DOCX)
