# Supplementary material for: Dietary patterns among adults in three low-income urban communities in Accra, Ghana
Source: PLoS One. 2023 Nov 9;18(11):e0293726. doi: 10.1371/journal.pone.0293726 (PMC10635542; doi:10.1371/journal.pone.0293726)

**Dietary patterns and quality among adults in three low-income urban communities in Accra, Ghana**

Sandra Boatemaa Kushitor^12*^, Deda Ogum Alangea^3^, Richmond Aryeetey^3^, and Ama de-Graft Aikins^4^

^1^Department of Food Science and Centre for Complex Systems in Transition, Stellenbosch University, Stellenbosch, South Africa

^2^Department of Community Health, Ensign College of Public Health, Kpong, Ghana

^3^Department of Population, Family and Reproductive Health, School of Public Health, University of Ghana, Legon-Accra, Ghana

^4^Institute of Advanced Studies, University College London, London, United Kingdom

## Supplementary File 1

| **Table S1. NYU-RIPS Twin Cities food frequency table for Ghanaians** | | | | | | | |
| --- | --- | --- | --- | --- | --- | --- | --- |
| **Food List** | **No. of times eaten in the last 7 days** | **Frequency of sources of food** | | | | | |
|  |  | **Home** | **Chop bar** | **Street vendor** | | **Fast food joint** | **Restaurant** |
| **CEREAL-BASED PORRIDGES** | | | | | | | |
| Millet koko (Housa Koko) |  |  |  |  | |  |  |
| Maize koko |  |  |  |  | |  |  |
| Ricewater |  |  |  |  | |  |  |
| Oats |  |  |  |  | |  |  |
| Other (specify)___________________ |  |  |  |  | |  |  |
| **CEREAL-BASED STAPLES** |  |  |  |  | |  |  |
| Rice-balls |  |  |  |  | |  |  |
| Banku/Akple/TZ/kenkey |  |  |  |  | |  |  |
| Plain rice |  |  |  |  | |  |  |
| Jollof rice |  |  |  |  | |  |  |
| Fried rice |  |  |  |  | |  |  |
| Waakye |  |  |  |  | |  |  |
| Other (specify)________________________ |  |  |  |  | |  |  |
| **TUBER & PLANTAIN BASED STAPLES** | | | | | | | |
| Fufu (all kinds) |  |  |  |  | |  |  |
| Ampesi (yam, cocoyam, plantain) |  |  |  |  | |  |  |
| Fried tubers (yam, plantain, cocoyam) |  |  |  |  | |  |  |
| Roasted tuber (yam, plantain, cocoyam |  |  |  |  | |  |  |
| Gari (*soakings, eba, or with beans*) |  |  |  |  | |  |  |
| Other (specify)___________________ |  |  |  |  | |  |  |
| **SOUPS** | | | | | | | |
| Palm soup |  |  |  |  | |  |  |
| Light soup |  |  |  |  | |  |  |
| Groundnut soup |  |  |  |  | |  |  |
| Kontomire soup |  |  |  |  | |  |  |
| Okro soup (fresh) |  |  |  |  | |  |  |
| Okro soup (dry) |  |  |  |  | |  |  |
| Other (specify)___________________ |  |  |  |  | |  |  |
| **STEWS/SAUCES** | | | | | | | |
| Tomatoes stew |  |  |  |  | |  |  |
| Palava sauce |  |  |  |  | |  |  |
| Garden egg stew |  |  |  |  | |  |  |
| Okro stew |  |  |  |  | |  |  |
| Beans stew *(with or without plantain)* |  |  |  |  | |  |  |
| Shito |  |  |  |  | |  |  |
| Ground pepper |  |  |  |  | |  |  |
| Other (specify)___________________ |  |  |  |  | |  |  |
| **FATS & OILS** | | | | | | | |
| Red palm oil |  |  |  |  |  | |  |
| Vegetable (frytol, soy bean) |  |  |  |  |  | |  |
| Butter |  |  |  |  |  | |  |
| Margarine |  |  |  |  |  | |  |
| Lard & animal fat |  |  |  |  |  | |  |
| Other (specify)___________________ |  |  |  |  |  | |  |
| **ANIMAL SOURCE FOODS** |  |  |  |  |  | |  |
| Livestock (goat, sheep, beef, pork) |  |  |  |  |  | |  |
| Poultry (chicken, duck, guinea fowl) |  |  |  |  |  | |  |
| Fish (tuna, herrings, salmon) |  |  |  |  |  | |  |
| Shell-fish (crab, lobster, shrimp, etc) |  |  |  |  |  | |  |
| Bush meat (grass cutter, antelope, etc) |  |  |  |  |  | |  |
| Egg |  |  |  |  |  | |  |
| Sausage |  |  |  |  |  | |  |
| Other (specify)___________________ |  |  |  |  |  | |  |
| **BAKED/ROASTED/BOILED SNACKS** |  |  |  |  |  | |  |
| Bran/wheat bread |  |  |  |  |  | |  |
| Sugar/tea/butter bread |  |  |  |  |  | |  |
| Meat pie |  |  |  |  |  | |  |
| Cakes |  |  |  |  |  | |  |
| Cashew nut |  |  |  |  |  | |  |
| Roasted groundnuts |  |  |  |  |  | |  |
| Roasted maize |  |  |  |  |  | |  |
| Other (specify)___________________ |  |  |  |  |  | |  |
| **FRIED SNACKS** |  |  |  |  |  | |  |
| Doughnut |  |  |  |  |  | |  |
| Chips (plantain , potato) |  |  |  |  |  | |  |
| Koose |  |  |  |  |  | |  |
| Chofi (Turkey tail) |  |  |  |  |  | |  |
| Other (specify)___________________ |  |  |  |  |  | |  |
| **SOFT DRINKS** |  |  |  |  |  | |  |
| Minerals (Fanta, Sprite coca cola) |  |  |  |  |  | |  |
| Malt drinks (Malta Guiness, vita malt) |  |  |  |  |  | |  |
| Fruit juices (Ceres, Pure heaven) |  |  |  |  |  | |  |
| Energy drinks (eg. Lucozade, Blue Jeans etc.) |  |  |  |  |  | |  |
| Sweetened (Tampico, Kalyppo ) |  |  |  |  |  | |  |
| Other (specify)___________________ |  |  |  |  |  | |  |
| **ALCOHOLIC DRINKS** |  |  |  |  |  | |  |
| Beers (Star, Club, Gulder)/Guinness |  |  |  |  |  | |  |
| Spirits (whiskey, gin) |  |  |  |  |  | |  |
| Wines |  |  |  |  |  | |  |
| Homebrews (palmwine, pito, akpeteshie) |  |  |  |  |  | |  |
| Bitters (e.g Alomo, Agya Appiah, etc) |  |  |  |  |  | |  |
| Other (specify)______________________ |  |  |  |  |  | |  |
| **MILK AND DAIRY PRODUCTS** |  |  |  |  |  | |  |
| Milk |  |  |  |  |  | |  |
| Yoghurt/Fanmilk |  |  |  |  |  | |  |
| Butter |  |  |  |  |  | |  |
| Cheese/Wagashie |  |  |  |  |  | |  |
| Other (specify)_______________________ |  |  |  |  |  | |  |
| **FRUITS** |  |  |  |  |  | |  |
| Orange |  |  |  |  |  | |  |
| Pineapple |  |  |  |  |  | |  |
| Banana |  |  |  |  |  | |  |
| Pawpaw |  |  |  |  |  | |  |
| Watermelon |  |  |  |  |  | |  |
| Mango |  |  |  |  |  | |  |
| Apple |  |  |  |  |  | |  |
| Grapes |  |  |  |  |  | |  |
| Avocado pear |  |  |  |  |  | |  |
| Coconut |  |  |  |  |  | |  |
| Other (specify)_______________________ |  |  |  |  |  | |  |
| **VEGETABLES** |  |  |  |  |  | |  |
| Green leafy vegetables(eg. kontomire, aleefu, ayoyo etc.) |  |  |  |  |  | |  |
| Cabbage |  |  |  |  |  | |  |
| Carrots |  |  |  |  |  | |  |
| Egg plant (all varieties) |  |  |  |  |  | |  |
| Okro |  |  |  |  |  | |  |
| Tomatoes |  |  |  |  |  | |  |
| Onion |  |  |  |  |  | |  |
| Other (specify)___________________ |  |  |  |  |  | |  |

**Table S2. Food groups and sub-items**

| **Food group** | **Food items** | **Frequency of consumption coded into** |
| --- | --- | --- |
| Animal source foods (ASF) | Livestock (goat, sheep, beef, pork), poultry (chicken, duck, guinea fowl), fish (tuna, herrings, salmon), shell-fish (crab, lobster, shrimp), bush meat (grasscutter, antelope, etc.) | <1x/wk  2-3x/wk  4-5x/wk  >5x/wk |
| Consumption of red meat | Livestock (goat, sheep, beef, pork | <1x/wk  2-3x/wk  4-5x/wk  >5x/wk |
| Consumption of fish and seafood | Fish (tuna, herrings, salmon), shell-fish (crab, lobster, shrimp) | <1x/wk  2-3x/wk  4-5x/wk  >5x/wk |
| Consumption of poultry | Poultry (chicken, duck, guinea fowl) | <1x/wk  2-3x/wk  4-5x/wk  >5x/wk |
| Consumption of processed ASF | Sausage | <1x/wk  2-3x/wk  4-5x/wk  >5x/wk |
| Consumption of eggs | Eggs | <1x/wk  2-3x/wk  4-5x/wk  >5x/wk |
| Fried snacks | Fried wheat flour chips, doughnuts | <1x/wk  2-3x/wk  4-5x/wk  >5x/wk |
| Baked snacks | Meat pie, cakes | <1x/wk  2-3x/wk  4-5x/wk  >5x/wk |
| Sugar sweetened beverages/ Fizzy drinks | Minerals (Fanta, Sprite, Coca cola), malt drinks (Malta Guiness, vita milk), sweetened drinks/fruit juices (Tampico, kalypo) | <1x/wk  2-3x/wk  4-5x/wk  >5x/wk |
| Energy drinks | Energy drinks (eg. Lucozade, Blue Jeans etc.) | <1x/wk  2-3x/wk  4-5x/wk  >5x/wk |
| Fruits | Orange, pineapple, banana, pawpaw, rice water, watermelon, mango, apple, grape, avocado, coconut |  |
| Vegetables | Green leafy vegetables, cabbage, carrots, egg plant, okro, tomatoes, onion | <1x/wk  2-3x/wk  4-5x/wk  >5x/wk |
| NOVA Group 1 unprocessed/minimally processed foods eg eggs, roasted corn-unsalted), | Millet koko, maize koko, rice water, oats, rice balls, banku, plain rice, waakye, fufu, roasted tuber, gari soakings, palm soup, light soup, groundnut soup, kontomire soup, grounded pepper, livestock, poultry, fish, shell-fish, bush meat, egg, roasted groundnuts, Roasted maize, cashew nut, egg, milk, orange, pineapple, banana, pawpaw, rice water, watermelon, mango, apple, grape, avocado, coconut, green leafy vegetables, cabbage, carrots, eggplant, okro, tomatoes, onion | <1x/wk  2-3x/wk  4-5x/wk  >5x/wk |
| NOVA Group 2 processed culinary ingredients eg salt, vegetable oil, honey, etc | Palm oil, vegetables oil, jollof rice, fried rice, fried tubers, tomatoes stew, palava sauce, garden egg stew, okro stew, shito, beans stews | <1x/wk  2-3x/wk  4-5x/wk  >5x/wk |
| NOVA Group 3 processed foods eg packaged, canned, bottled fruit, vegetable, legume, fish, breads, cheese etc | Bran bread, sugar bread, meat pie, cakes, doughnuts, chips, cheese, butter, margarine | <1x/wk  2-3x/wk  4-5x/wk  >5x/wk |
| NOVA Group 4 ultra-processed industrially formulated foods and drinks eg chocolate, pizza, sausage, noodle, yoghurt, etc. | Minerals (fizzy drinks), malt drinks, fruit juice, energy drinks, sweetened drinks, sausage, yoghurt/ice cream | <1x/wk  2-3x/wk  4-5x/wk  >5x/wk |

**Table S3.** **Factor loadings of food items in the EDULINK, 2011 and 2013**

| **Food Items** | **Snack pattern** | **Staple and sauce pattern** | **Rice pattern** |
| --- | --- | --- | --- |
| Millet_Koko | 0.0512 | 0.1105 | 0.0052 |
| Riceball | 0.1323 | 0.2708 | -0.0096 |
| Kenkey | -0.0812 | 0.132 | 0.3014 |
| Plainrice | 0.1361 | -0.0925 | 0.5653 |
| Fried rice | 0.2595 | 0.1588 | 0.2574 |
| Jollof | 0.2747 | 0.0928 | 0.3757 |
| Fufu | 0.0161 | 0.4463 | 0.1033 |
| Ampesi | 0.0388 | 0.3865 | 0.0950 |
| Fried tubers | 0.2212 | 0.1939 | 0.1311 |
| Roastedtub~S | 0.2200 | 0.1506 | 0.0757 |
| Gari | 0.1324 | 0.1981 | 0.1565 |
| Palmsoup | 0.0019 | 0.4284 | 0.0948 |
| Lightsoup | 0.0178 | 0.4628 | 0.1845 |
| Gnutsoup | 0.0749 | 0.3418 | 0.0967 |
| Kontsoup | 0.1215 | 0.3473 | 0.0118 |
| Okrosoup fresh | 0.0506 | 0.2817 | 0.1793 |
| Okrosoup dry | 0.0713 | 0.1834 | 0.0129 |
| Tomatoes stew | 0.0828 | -0.0114 | 0.6294 |
| Palava sause | 0.0694 | 0.3869 | 0.0684 |
| Garden egg stew | 0.0463 | 0.4375 | 0.1365 |
| Okro stew | 0.0611 | 0.3022 | 0.1885 |
| Bean stew | 0.1667 | 0.2205 | 0.1045 |
| Shito | 0.1106 | 0.1502 | 0.4125 |
| Grounded pepper | 0.0141 | 0.1793 | 0.3364 |
| Palm oil | 0.0354 | 0.3981 | 0.2522 |
| Frytol | 0.1588 | 0.0325 | 0.5334 |
| Cake | 0.4997 | 0.1321 | -0.0077 |
| Meat pie | 0.4837 | 0.1155 | 0.1064 |
| Chips | 0.4724 | 0.126 | -0.0293 |
| Doughnut | 0.4412 | 0.1162 | -0.0253 |
| Fruit juice | 0.3951 | 0.1333 | 0.1113 |
| Malt | 0.3944 | 0.1807 | 0.0964 |
| Frozen diary (e.g Fanmilk, Fanyoghurt) | 0.3888 | 0.1458 | 0.1702 |
| Soda | 0.3454 | 0.0581 | 0.2938 |
| Fruit flavoured drinks (e.g Tampico) | 0.3316 | 0.1643 | 0.1083 |
| Sausage | 0.3240 | 0.0004 | 0.2038 |
| Banana | 0.3089 | 0.2838 | 0.0296 |
| Milk | 0.3016 | 0.0205 | 0.18 |
| Egg | 0.2919 | 0.0355 | 0.2936 |
| Margarine | 0.2913 | -0.0362 | 0.1708 |
| Roasted groundnut | 0.2838 | 0.1792 | 0.1507 |
| Grapes | 0.2813 | 0.099 | -0.0319 |
| Avocado | 0.2804 | 0.1742 | -0.0142 |
| Sugar bread | 0.2782 | 0.0453 | 0.2526 |
| Butter | 0.2708 | -0.0281 | 0.0890 |
| Chofi | 0.2654 | 0.211 | 0.1196 |
| Energydrink | 0.2449 | 0.1243 | 0.1313 |
| Poultry | 0.2401 | 0.134 | 0.295 |
| Roasted maize | 0.2310 | 0.2118 | 0.0625 |
| Watermelon | 0.2272 | 0.3582 | -0.0256 |
| Pineapple | 0.2193 | 0.3357 | 0.0145 |
| Koose | 0.1989 | 0.2341 | 0.0486 |
| Orange | 0.1964 | 0.2440 | 0.0313 |
| Pawpaw | 0.1951 | 0.3659 | -0.0106 |
| Wheat bread | 0.1882 | 0.0781 | 0.0028 |
| Cashew nut | 0.1229 | 0.0685 | 0.0091 |
| Cheese | 0.0935 | -0.0065 | 0.0054 |
| Livestock | 0.0897 | 0.1833 | 0.3281 |
| Shellfish | 0.0813 | 0.2634 | 0.1376 |
| Lard | 0.0564 | 0.0267 | 0.0564 |
| Bushmeat | 0.0503 | 0.1754 | -0.024 |
| Fish | -0.0715 | 0.2046 | 0.3415 |

**Fig S1. Scree plot of eigenvalues after factor**


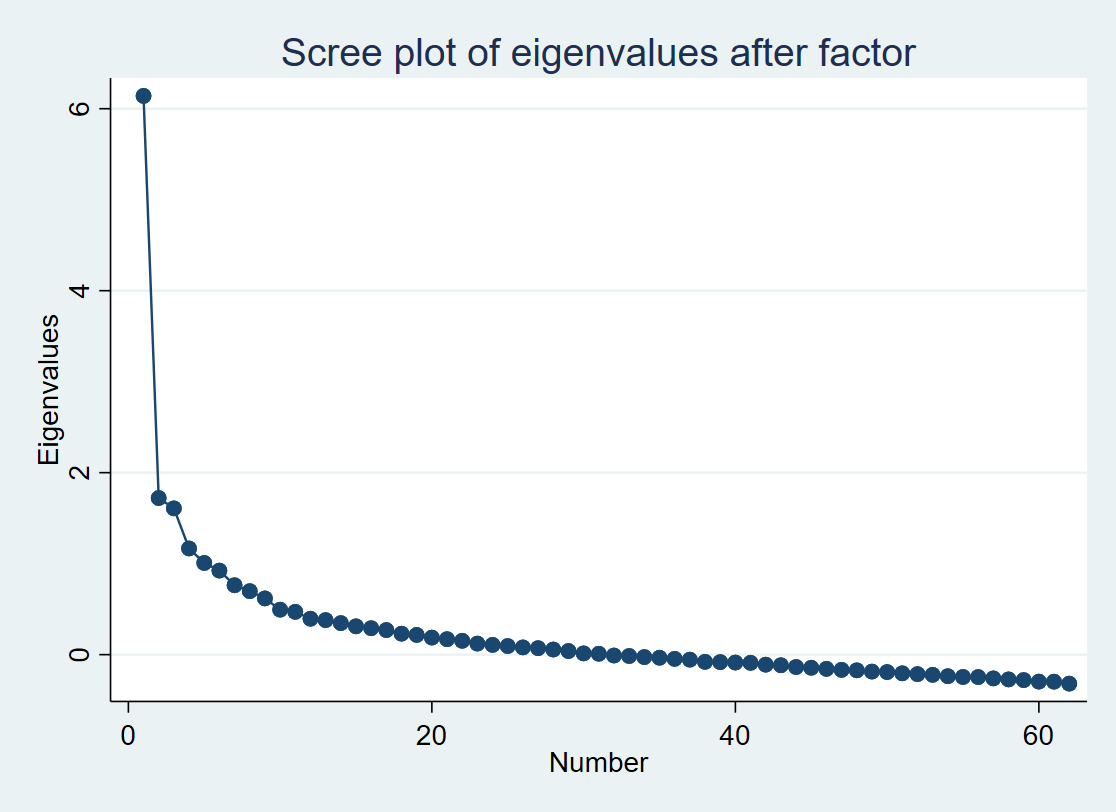

Supplement: S1 File — (DOCX) [file pone.0293726.s001.docx]
